# Dual-tracer PET/CT imaging with ^68^Ga-DOTATATE and Al^18^F-NOTA-FAPI for dynamic evaluation of myocardial inflammation and fibrosis in antimitochondrial antibody-positive myositis

**DOI:** 10.1515/rir-2026-0003

**Published:** 2026-03-30

**Authors:** Junyan Qian, Jiqing Chen, Xinyan Wen, Siqi Tang, Na Niu, Huaxia Yang

**Affiliations:** Department of Rheumatology and Clinical Immunology, Peking Union Medical College Hospital, Chinese Academy of Medical Sciences, Peking Union Medical College, Beijing, China; National Clinical Research Center for Rheumatic and Autoimmune Diseases (NCRC-RAD), National Health Commission, Beijing, China; State Key Laboratory of Complex Severe and Rare Diseases, Peking Union Medical College Hospital, Ministry of Science & Technology, Beijing, China; Key Laboratory of Rheumatology and Clinical Immunology, Ministry of Education, Beijing, China; Key Laboratory of Intelligence and Precision in Rheumatic and Autoimmune Diseases, Beijing Municipal Commission of Science & Technology, Beijing, China; Department of Rheumatology and Clinical Immunology, South-East University affiliated Zhongda Hospital, Nanjing, China; Department of Internal Medicine, Peking Union Medical College Hospital, Chinese Academy of Medical Sciences and Peking Union Medical College, Beijing, China; Department of Nuclear Medicine, Peking Union Medical College Hospital, Chinese Academy of Medical Sciences and Peking Union Medical College, Beijing, China; Beijing Key Laboratory of Molecular Targeted Diagnosis and Therapy in Nuclear Medicine, Beijing, China

A 36-year-old man was presented with one-year history of progressive exertional chest tightness, dyspnea, and palpitation. Initial evaluation revealed a positive antinuclear antibody (ANA) with a cytoplasmic pattern at a titer of 1: 80, alongside positivity AMA-M2, and SP100. Liver function tests showed notably elevated gamma-glutamyl transferase (GGT) at 986 U/L, alkaline phosphatase (ALP) at 266 U/L, and mildly elevated transaminases. Creatine kinase (CK), CK-MB, high-sensitivity troponin I (hs-TnI), and N-terminal pro-brain natriuretic peptide (NT-proBNP) were within normal limits. However, echocardiography demonstrated significant structural and functional abnormalities, including biatrial enlargement, left ventricular dilation, and a reduced left ventricular ejection fraction (LVEF) of 40%. Diffuse left ventricular wall hypokinesia and endocardial hyperechogenicity were also observed. Furthermore, 24-h ambulatory electrocardiogram (ECG) monitoring documented frequent atrial and ventricular premature contractions, as well as intermittent atrioventricular blocks. Based on these findings, the patient was diagnosed with primary biliary cholangitis (PBC), connective tissue disease, and AMA-M2-positive myositis.

**Figure 1:**
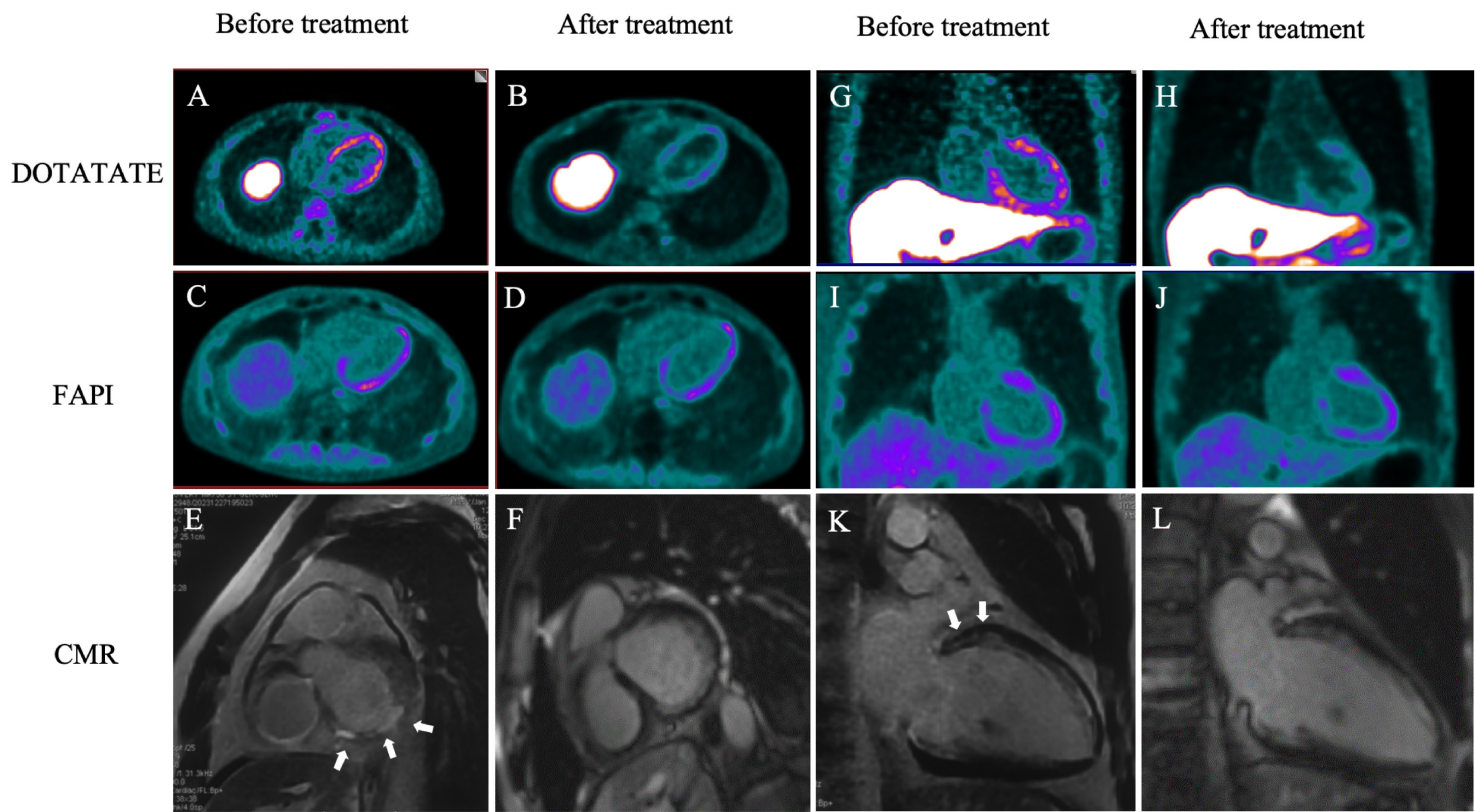
Longitudinal imaging assessment of myocardial inflammation and fibrosis before and after immunosuppressive therapy. (A, B, G, H) ^68^Ga-DOTATATE PET/CT images. Baseline imaging (A, G) demonstrates diffuse myocardial uptake in the interventricular septum and left ventricular wall (SUVmax 4.04), indicating macrophage-driven inflammation. Post-treatment (B, H), this uptake is significantly reduced (SUVmax 2.51), consistent with resolved inflammation. (C, D, I, J) Al18F-NOTA-FAPI-04 PET/CT images. Baseline (C, I) shows focal, heterogeneous uptake in the anterior, lateral, and inferior walls (SUVmax 3.79), suggestive of fibroblast activation. Post-treatment (D, J), the focal uptake persists with a slight increase in SUVmax (4.18), indicating residual fibrotic burden. (E, F, K, L) Cardiac magnetic resonance (CMR) with late gadolinium enhancement (LGE). Baseline short-axis (E) and two-chamber long-axis (K) views show patchy mid-wall LGE (arrows), indicative of active inflammation or fibrosis. Follow-up CMR (F, L) after therapy shows partial resolution of the LGE (arrows).

The patient was initiated on a 6-month regimen of high-dose methylprednisolone (1 g/d intravenously for 3 days, followed by a gradual taper to 6 mg/d orally) combined with mycophenolate mofetil (0.75 g twice daily). The patient was treated with ursodeoxycholic acid (UDCA) at a standard dose of 250 mg three times daily. Following this treatment, liver function tests normalized, and serum myocardial markers remained within normal limits, which was accompanied by significant symptomatic improvement, including the resolution of dyspnea and normalization of the LVEF to 60%. To dynamically assess myocardial inflammation and fibrosis, dual-tracer positron emission tomography/computed tomography (PET/CT) imaging with ^68^Ga-DOTATATE and Al^18^F-NOTA-FAPI-04 was performed at baseline and after immunosuppressive therapy, in conjunction with cardiac magnetic resonance (Figure 1). Baseline ^68^Ga-DOTATATE PET revealed diffuse myocardial uptake, which markedly resolved after therapy, paralleling the improvement in cardiac function. In contrast, Al^18^F-NOTA-FAPI-04 PET demonstrated focal uptake that persisted despite treatment.

The presence of anti-mitochondrial M2 antibody (AMA-M2), a serological hallmark of PBC, is also identified in 2.5%-19.5% of myositis patients, suggested potential immune-mediated cardiac involvement in this case.^[1, 2, 3, 4, 5]^ This case collectively underscores the complementary roles of ^68^Ga-DOTATATE and Al^18^F-NOTA-FAPI-04 PET/CT for staging immune-mediated myocardial injury, with the former mapping inflammatory activity and the latter quantifying the fibrotic burden. The observed temporal dissociation, wherein inflammatory resolution (reflected by the decline in DOTATATE signal) is decoupled from fibrotic evolution (indicated by persistent fibroblast activation protein inhibitor [FAPI] uptake), highlights the unique potential of this dual-tracer approach for monitoring therapeutic efficacy and predicting disease progression. In conclusion, the complementary application of ^68^Ga-DOTATATE for inflammation and Al^18^F-NOTA-FAPI-04 for fibrosis provides a powerful non-invasive tool for diagnosing, risk-stratifying, and therapeutically monitoring cardiac involvement in patients with AMA-positive myositis.
